# Patterns of Transmitted Drug Resistance Mutations and HIV-1 Subtype Dynamics in ART-Naïve Individuals in Veneto, Italy, from 2017 to 2024

**DOI:** 10.3390/v16091393

**Published:** 2024-08-30

**Authors:** Nicholas Geremia, Monica Basso, Andrea De Vito, Renzo Scaggiante, Mario Giobbia, Giuliana Battagin, Federico Dal Bello, Maria Teresa Giordani, Stefano Nardi, Marina Malena, Annamaria Cattelan, Saverio Giuseppe Parisi

**Affiliations:** 1 Unit of Infectious Diseases, Department of Clinical Medicine, Ospedale Dell’Angelo, 30174 Venice, Italy; nicholas.geremia@aulss3.veneto.it; 2 Unit of Infectious Diseases, Department of Clinical Medicine, Ospedale Civile “S.S. Giovanni e Paolo”, 30122 Venice, Italy; 3 Department of Molecular Medicine, University of Padua, 35121 Padua, Italy; monica.basso@unipd.it (M.B.); federico.dalbello@unipd.it (F.D.B.); annamaria.cattelan@unipd.it (A.C.); saverio.parisi@unipd.it (S.G.P.); 4 Unit of Infectious Diseases, Department of Medicine, Surgery and Pharmacy, University of Sassari, 07100 Sassari, Italy; 5 Biomedical Science Department, School in Biomedical Science, University of Sassari, 07100 Sassari, Italy; 6 Unit of Infectious Diseases, AULSS 1, 32100 Belluno, Italy; renzo.scaggiante@aulss1.veneto.it; 7 Unit of Infectious Diseases, AULSS 2, 35100 Treviso, Italy; mario.giobbia@aulss2.veneto.it; 8 Unit of Infectious Diseases, AULSS 8, 36100 Vicenza, Italy; giuliana.battagin@aulss8.veneto.it; 9 Unit of Infectious Diseases, AULSS 7, 36014 Santorso, Italy; mariateresa.giordani@aulss7.veneto.it; 10 Unit of Infectious Diseases, AULSS 9, 37122 Verona, Italy; stefano.nardi@aulss9.veneto.it; 11 Unit of Infectious Diseases, AULSS 5, 45100 Rovigo, Italy; marina.malena@aulss5.veneto.it

**Keywords:** HIV, genotypic resistance testing, transmitted drug resistance mutations, HIV subtypes, HIV resistance

## Abstract

This study investigates the prevalence and patterns of transmitted drug resistance mutations (TDRMs) and HIV-1 subtypes among antiretroviral therapy (ART) naïve individuals in Veneto, Italy, from 2017 to 2024. This research aims to understand the dynamic landscape of TDRMs and HIV-1 genetic diversity to inform treatment strategies effectively. We included all adult ART-naïve people with HIV (PWH) from seven infectious disease units in Veneto, Italy. We collected the genotypic resistance testing conducted to predict drug susceptibility and subtype distribution using the Stanford HIVdb algorithm. We included 762 PWH, showing a slight but statistically significant decline in the B subtype among Italian PWH (*p* = 0.045) and an increase in non-B subtypes among foreigners, though it was not statistically significant (*p* = 0.333). The most frequent mutations were in Non-nucleoside Reverse Transcriptase Inhibitors (NNRTIs), especially in non-B subtypes, with a notable rise from 10.7% in 2017–2019 to 15.5% in 2020–2024. Notably, TDRMs were consistently detected, highlighting an ongoing challenge despite the stable prevalence observed over the years. In addition, the data revealed a concerning rise in mutations against newer drug classes, such as integrase inhibitors. Conclusively, the study underscores the necessity of continuous surveillance of HIV subtypes and resistance patterns to adapt ART regimens optimally. Despite the stable levels of drug resistance, the emergence of resistance against newer drugs necessitates ongoing vigilance and possible adjustment in treatment protocols to enhance clinical outcomes and manage HIV drug resistance effectively.

## 1. Introduction

Antiretroviral therapy (ART) has brought about a fundamental change in the management of HIV infections, leading to a period of longer longevity and significantly lower morbidity rates for persons living with HIV (PWH) [1,2]. Nonetheless, the appearance and spread of transferred drug resistance mutations (TDRMs) in PWH who have not yet started ART continue to be major issues that could jeopardize the effectiveness of first treatment plans and present significant obstacles to the worldwide management of HIV infection [3].

Recent guidelines advocate for the prompt initiation of ART, irrespective of CD4+ cell count, to mitigate the risk of HIV transmission and progression to AIDS [4,5]. Nonetheless, the rapid implementation of ART is contingent upon a nuanced understanding of the prevalence and patterns of TDRMs, as well as the genetic diversity of circulating HIV-1 subtypes, which may influence the selection of antiretroviral drugs and the subsequent risk of treatment failure.

Studies conducted within distinct cohorts in Italy have clarified the dynamics of TDRMs and the HIV-1 subtypes epidemiology. For instance, an investigation into the Veneto region’s HIV-infected population from 2004 to 2012 revealed a declining trend in the prevalence of TDRMs among individuals infected with subtype B strains, albeit with a concomitant rise in non-B subtype infections [6]. This finding underscores the intricate interplay between HIV-1 genetic diversity and the landscape of drug resistance, necessitating comprehensive surveillance to inform ART initiation strategies effectively.

Furthermore, analysis of antiretroviral therapy-naïve PWH attending Sapienza University Hospital in Rome from 2006 to 2017 unveiled a substantial presence of various HIV-1 subtypes and circulating recombinant forms (CRFs), with subtype B predominating [7]. Notably, this period also marked the identification of major integrase strand transfer inhibitor (INSTI) resistance mutations in ART-naïve individuals for the first time in Italy, highlighting the evolving challenge posed by TDRMs to newer ART classes and underscoring the stable prevalence of TDRMs over time.

In light of these observations, our study aims to comprehensively analyze the patterns of TDRMs, the distribution of HIV-1 subtypes, and the rates of late presentation among treatment-naïve individuals diagnosed with HIV infection over the last seven years. This analysis is conducted during two distinct periods, 2017–2019 (pre-COVID-19) and 2020–2024 (post-COVID-19), to assess the potential impact of the COVID-19 pandemic as well as ongoing epidemiological trends. By examining genotypic resistance testing (GRT) data across these periods, we aim to provide insights into the current dynamics of TDRMs, subtype diversity, and late presentations, which are crucial for optimizing antiretroviral therapy (ART) strategies and improving clinical outcomes.

## 2. Materials and Methods

### 2.1. Study Design

Adults living with HIV ART-naïve were included in the study at the time of HIV infection diagnosis in seven infectious disease units in Veneto, Italy, from 1 January 2017 to 30 April 2024. Eligibility criteria included age > 18 years and antiretroviral drug-naive status according to a personal interview and a review of the history of infection since the first positive serological test.

We decided to divide the study period into 2017–2019 and 2020–2024 to analyze potential temporal evolution in our data. A longer period would likely obscure any significant trends or changes. The division of these periods also reflects the impact of the COVID-19 pandemic, which introduced substantial variations in patient enrollment due to lockdowns and the closure of clinics. Thus, comparing these two distinct periods allows us to better understand how these external factors influenced our study’s outcomes.

### 2.2. Immunovirological Analysis

Blood samples were processed within six hours of collection at the Laboratory of Virology, University Hospital of Padova, Italy, and subsequently stored until analyzed. A primary or recent HIV infection was defined by one of the following criteria: (I) an enzyme-linked immunosorbent assay (ELISA) showing negative or indeterminate HIV antibodies coupled with a positive plasma HIV RNA test, or (II) an initially negative HIV antibody test followed by positive serology within 18 months.

At the time of sampling, the absolute count and percentage of CD4+ cells, as well as the HIV-RNA plasma viral load, were measured. However, HIV-RNA values were not included in the statistical analysis due to the use of various commercial methods with differing sensitivities throughout the observational period, which rendered the data incomparable. Genotypic Resistance Testing (GRT) results from this study were compared with data from previous surveys conducted during 2004–2012 and 2013–2016, adhering to the same inclusion criteria. The ViroSeq HIV-1 Genotyping System by Celera Diagnostics, Alameda, CA, USA, was employed for genotyping and identifying mutations.

### 2.3. Prediction of Susceptibility and Subtype Analysis

The Stanford HIVdb drug resistance algorithm version 9.6 was utilized to evaluate the potential impact of transmitted drug resistance mutations (TDRMs) on therapeutic responses. This algorithm assigns a specific score to each detected TDRM. The cumulative score from all TDRMs identified in a single viral strain categorizes it into one of five susceptibility levels: susceptible (S), potential low-level resistance (PLR), low-level resistance (LR), intermediate resistance (IR), and high-level resistance (HR). A designation of reduced susceptibility was assigned when the Stanford system identified resistance at a minimum of low-level resistance (LR), thus focusing on clinically significant resistance patterns. Additionally, subtyping of HIV-1 genetic sequences was conducted using the automated tools provided by Stanford HIVdB and the REGA Institute Subtyping tool version 3.0 for both clinical and surveillance purposes.

### 2.4. Statistical Analysis

Data were collected with Excel (Microsoft, Redmond, WA, USA). Data were described using mean and standard deviation (SD) for normally distributed continuous variables, median and interquartile range (IQR) for not normally distributed continuous variables, and frequency (%) for categorical and ordinal variables. The normality of distribution was assessed using the Shapiro–Wilk test. Differences between the time periods were evaluated using the chi-square test or Fisher exact test, as appropriate. Statistical significance was set at *p*-values of less than 0.05, and data analysis was carried out through STATA (Version 17 StataCorp, College Station, TX, USA).

### 2.5. Ethical Issues

The study was approved by the Ethical Committee for Clinical Experimentation, Padua Province (Ethics Committee Protocol no. 2606-12P). The subjects gave informed consent for all procedures and for using their blinded data for scientific evaluation and publication. This study was conducted in accordance with the Helsinki Declaration and local legislation.

## 3. Results

### 3.1. Demographical, Immunological, and Virological Characteristics

We included 762 PWH, 414 from 1 January 2017 to 31 December 2019, and 348 from 1 January 2020 to 30 April 2024. The median age was 40 (IQR31–48.5) years. The majority of them were Italian (470, 61.7%) and assigned male at birth (569, 74.7%).

B and non-B subtypes were 227 (54.8%) and 187 (45.2%), respectively, in 2017–2019, and 167 (48.0%) and 181 (52.0%) in 2020–2024. From 2017 to 2024, there was a slight decline in the B subtype population (from 54.8% to 48.0%) and an increased prevalence of non-B subtypes (from 45.2% to 52.0%), which was not statistically significant (*p*-value = 0.059). Among PWH of Italian origin, the subtype B was the most prevalent, with 191 (74.9%) in 2017–2019 and 143 (66.5%) in 2020–2024. The decrease in subtype B among Italian PWH was statistically significant (*p*-value 0.045). On the contrary, we observed non-B subtypes to be the most prevalent in PWH with foreign origin, with 123 (77.4%) in 2017–2019 and 109 (81.9) in 2020–2024. The slight increase in non-B subtypes between 2017–2019 and 2020–2024 was not statistically significant (*p*-value = 0.333).

Regarding sex, males were most frequently infected with the B subtype, with 193 (61.8%) in 2017–2019 and 137 (53.3%) in 2020–2024, showing a statistically significant decrease between the two time points (*p*-value 0.028). This decrease was primarily due to a reduction in PWH of Italian origin, where the percentage of subtype B decreased from 76.3% to 67.8% between 2017–2019 and 2020–2024.

On the contrary, the most frequently observed HIV subtypes in females were those other than subtype B, with a prevalence of 68.3% in 2017–2019 and 67.0% in 2020–2024. ART-naïve females with HIV were predominantly of foreign origin. Females born in Italy were 31 (30.4%) in 2017–2019 and 41 (45.0%) in 2020–2024. The most common subtype detected in Italian females was B, which was detected in 64.5% of Italian females in 2017–2019 and 60.9% in 2020–2024.

Genotype and demographical characteristics are summarized in Table 1.

**Figure 1:**
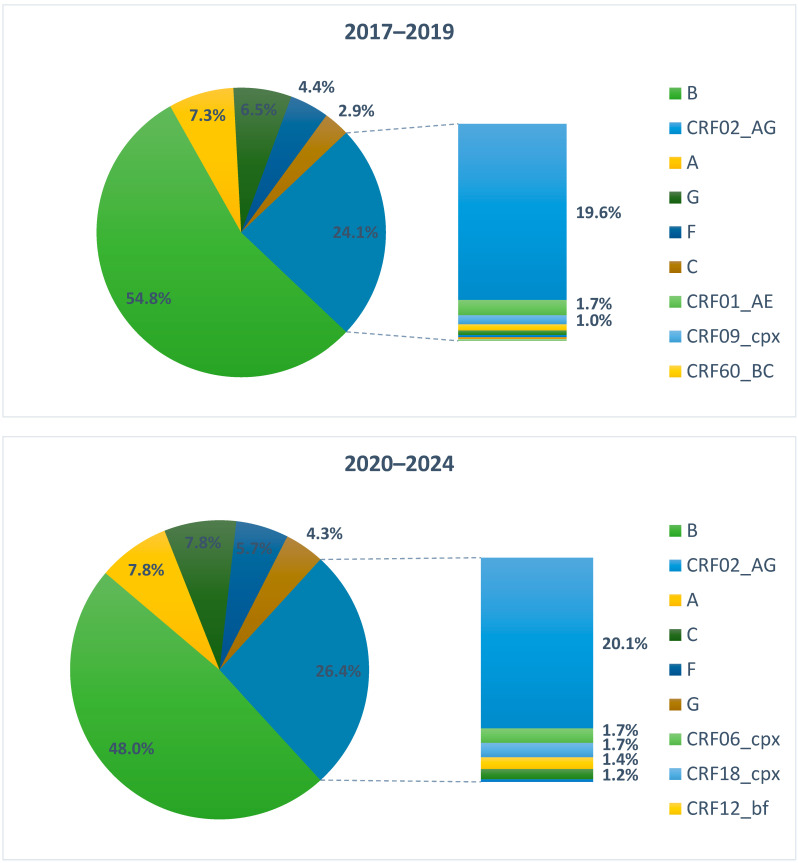
B and Non-B HIV-1 clades identified in 762 naïve people with HIV and divided into 2017–2019 and 2020–2024. Percentages < 1% were not reported.

Regarding the non-B subtypes, we observed an increase in the two periods. In 2017–2019 and 2020–2024), they were 46.2%, while in 2020–2024, the non-B subtypes overcame the B subtypes, with a prevalence of 51.2%. Looking inside the non-B subtypes, the most common form detected was the Circulating Recombinant Forms (CRF), which were 24.1% in 2017–2019 and 26.4% in 2020–2024. We also observed an increase in the C and F subtypes; on the contrary, we observed a reduction in the G subtype, while the A subtype remained stable (Figure 1).

Regarding the viro-immunological data at the diagnosis, the median of the HIV viral load in 2017–2019 was similar to B and non-B subtypes (86,200 cp/mL vs. 86,691 cp/mL. Instead, in 2020–2024, the viral load was 109,000 cp/mL for the B subtype population and 140,309 cp/mL for non-B subtypes. Regarding the immunological status, in 2017–2019, the baseline median of CD4+ absolute cells count was 277 cells/mm^3^ (CD4% 16.7%) for the B subtype and 294 cells/mm^3^ (CD4% 16.0%) for the non-B subtype. In 2020–2024, CD4+ absolute counts were 248 cells/mm^3^ (CD4% 18.0%) and 208 cells/mm^3^ (CD4% 16.5%) for the B and the non-B subtypes, respectively. Most in detail, we observed a high percentage of late presenters, which remains stable in people with non-B subtypes and increases in people with B-subtype. In addition, we observed an increase in people with less than 200 CD4 cells/mm^3^ in the two periods. In people with B-subtype, the percentage increased from 39% to the percentage of people with less than 200 CD4 cells/mm^3^ was 39.0% and 39.1% for the B and non-B subtypes in 2017–2019, and 45.8% and 46.7% for the B and the non-B subtypes, respectively. Immunitary and virological data are shown in Table 2.

### 3.2. Drug Susceptibility Prediction

Fifty-seven PWH (13.7%) in 2017–2019, and 59 PWH (16.5%) were resistant to at least one drug. The most frequent single-class mutations were observed in the Non-nucleoside Reverse Transcriptase Inhibitors (NNRTI) class. The B subtype presented NNRTI TDRMs in 25 cases (11.0%) in 2017–2019 and 17 cases (10.2%) in 2020–2024. For non-B subtypes, NNRTI mutations were observed in 20 cases (10.7%) in 2017–2019 and 28 cases (15.5%) in 2020–2024. In B subtypes, the second most frequent TDRMs were found in NRTIs (7/227, 3.0% in 2017–2019 and 4/167, 2.4%).

Drug susceptibility prediction is described in Table 3.

We performed a subanalysis, dividing the resistance genotype by PWH origin. In this period, the NNRTI DRMs were most prevalent in extra-Europe origin (17.5%). Instead, it was similar between Italians and other Europeans (13.9% vs. 13.8%). Even with the low proportion of cases, N + NNRTI DRMs were observed only in foreigners (one case for other Europeans and two cases for extra Europeans). The data are summarised in Table 4 and Table 5.

**Figure 2:**
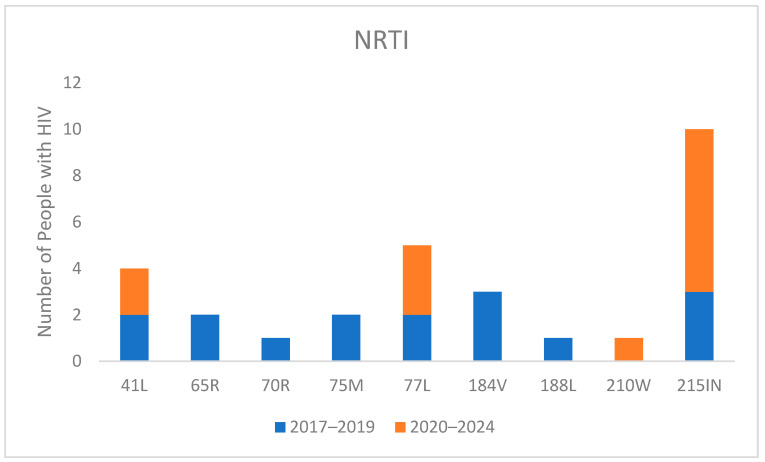
Mutations for Nucleoside Reverse Transcriptase Inhibitor (NRTI) in naive people with HIV divided into 2017–2019 and 2020–2024 periods.

Regarding the specific mutations, as shown in Figure 2 for NRTI-related changes, the most common mutation was T215IN. This revertant mutation suggests that the patient may have once been infected with a virus containing T215Y/F. It was present in three individuals during 2017–2019 and increased to seven individuals in 2020–2024. M184V was detected in only three individuals in 2017–2019, while no patients exhibited this mutation in the period from 2020 to 2024. Similarly, K65R was present in only two people in 2017–2019 and was not observed in anyone from 2020–2024.

Regarding NNRTI-associated mutations, as shown in Figure 2, the most common mutation was E138AK, which reduces rilpivirine (RPV) susceptibility two to three-fold. It was present in 6.3% of individuals in 2017–2019 and slightly increased to 6.8% of individuals in 2020–2024. Another significant mutation, K103N, which confers high-level reductions in susceptibility to nevirapine and efavirenz, was consistently present in ten individuals in both time periods.

**Figure 3:**
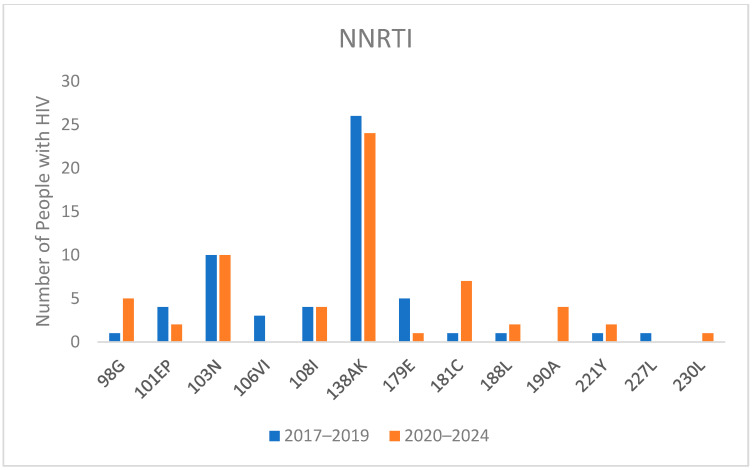
Mutations for Non-nucleoside Reverse Transcriptase Inhibitor (NNRTI) in naive people with HIV divided into 2017–2019 and 2020–2024 periods.

Noteworthy, mutations Y188L, F227L, and M230L, which confer resistance to doravirine and RPV, were identified in three, one, and one individual respectively (Figure 3).

**Figure 4:**
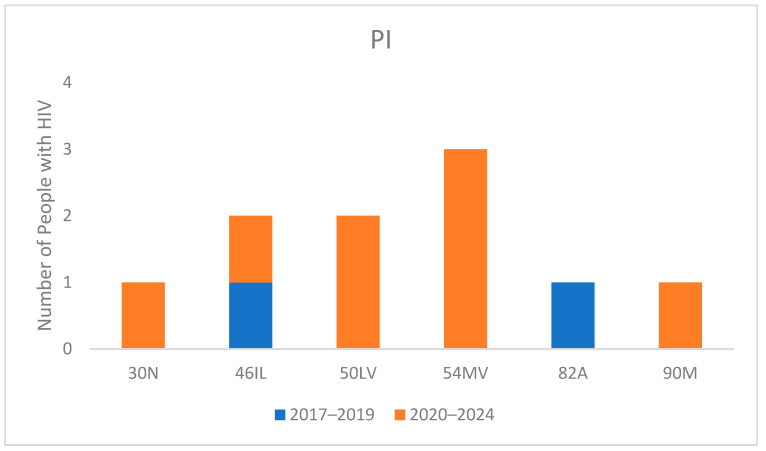
Mutations for Protease inhibitors (PI) in naive people with HIV divided into 2017–2019 and 2020–2024 periods.

Protease-inhibitor-related mutations (Figure 4) showed minimal occurrences. The most common mutation, I54MV, a non-polymorphic mutation selected primarily by fosamprenavir and darunavir (DRV), reduces susceptibility to lopinavir, atazanavir, and DRV.

**Figure 5:**
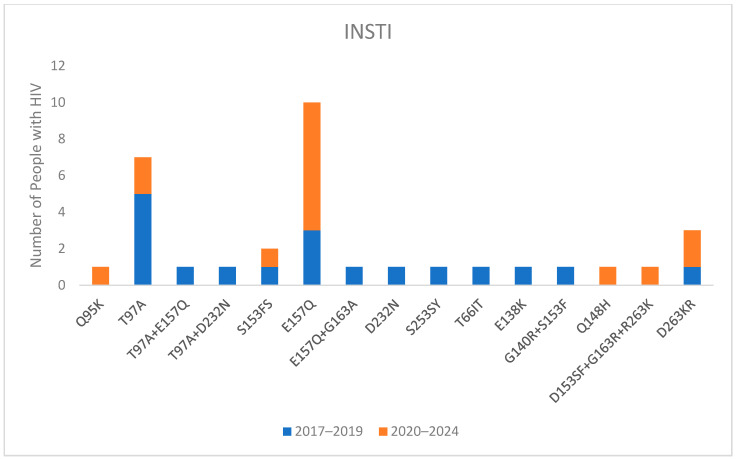
Mutations for integrase in naive people with HIV divided into 2017–2019 and 2020–2024 periods.

The integrase GRT was not available for everyone. Specifically, only 293 out of 414 people (70.8%) had a GRT on integrase in 2017–2019, and 283 out of 348 (81.3%) had a GRT on integrase in 2020–2024. Among these, 28 out of 293 (7.1%) and 19 out of 283 (6.7%) had detectable mutations (Figure 5). However, major mutations were observed in only 8 out of 576 people (1.4%). Additionally, resistance to bictegravir (BIC) and dolutegravir (DTG) was observed in only six (1.0%) PWH: one with the presence of D153SF + G163R + R263K, one with Q148H, one with G140R + S153F, and three with D263KR.

## 4. Discussion

Modern ART regiments have profoundly modified the natural history of PWH [1,8]. However, TDRMs in ART-naïve PWH could compromise the efficacy of initial treatment regimens. Additionally, globalization and migration patterns can influence the spread of HIV polymorphisms. Notably, Mediterranean countries have become significant destinations for migrants from sub-Saharan Africa, South America, and Eastern Europe over the past decade. This influx has facilitated the dissemination of diverse viral variants, each with unique polymorphisms and resistance pathways [9].

Our study provides a thorough analysis of the prevalence and trends of TDRMs and HIV-1 subtypes in a cohort of ART-naïve individuals in Veneto, Italy, focusing on two distinct periods: 2017–2019 (pre-COVID-19) and 2020–2024 (post-COVID-19). The data reveal a complex picture of HIV subtype distribution and resistance, reflecting both global trends and the potential influence of the COVID-19 pandemic on these dynamics. These findings have significant implications for ART strategies, especially in understanding the pandemic’s impact on HIV treatment and management. The COVID-19 pandemic has had a significant impact on HIV diagnosis, as healthcare systems were overwhelmed and resources redirected towards managing the pandemic. A multicentric Italian study reported a substantial reduction in HIV diagnoses during the pandemic, probably due to social distancing measures and reduced access to testing services. This shift underscores the need for robust HIV screening and diagnostic strategies, particularly during public health crises, to avoid missed diagnoses and late presentations [10]. In line with these findings, our study also observed a noticeable reduction in HIV diagnoses during the years 2020 and 2021, as shown in Supplementary Table S1. This decrease likely reflects the impact of the COVID-19 pandemic on routine HIV screening and diagnostic activities, as healthcare resources were redirected and access to testing was limited.

Our findings on the shift from HIV-1 subtype B to non-B subtypes in Veneto from 2017 to 2024 reflect broader trends previously documented in the region. The decrease in subtype B from 54.8% to 48.0% aligns with an earlier decline from 67.3% in 2013–2016. Concurrently, the increase in non-B subtypes from 45.2% to 52.0% aligns with growth from 21.9% to 33.0% between 2004 and 2012 [6,11].

The shift from HIV-1 subtype B to non-B subtypes in Veneto from 2017 to 2024 reflects broader trends previously documented in the region. This ongoing diversification of HIV-1 subtypes is likely influenced by international mobility and migration. Veneto, like other Mediterranean regions, has seen substantial migration from sub-Saharan Africa, South America, and Eastern Europe [12,13]. These regions have a higher prevalence of non-B subtypes, which are being introduced into the local population. The decrease in subtype B among Italian-origin PWH may reflect changing social and sexual networks that include individuals from diverse geographic backgrounds. This trend is particularly evident in males, where the reduction in subtype B prevalence may indicate increased interactions with individuals who predominantly carry non-B subtypes.

Conversely, the prevalence of non-B subtypes among foreign-origin PWH remains high, consistent with their regions of origin where these subtypes are more common. This persistence suggests that while these individuals may bring diverse HIV strains into the area, they continue to circulate predominantly within their communities. Though not statistically significant, the slight increase in non-B subtypes among foreign-origin PWH between 2017–2019 and 2020–2024 aligns with the continued influx of migrants from regions with high non-B subtype prevalence. We observed a high percentage of CRF02_AG, with 19.6% in 2017–2019 and 20.1% in 2020–2024. This subtype is prevalent in many regions of sub-Saharan Africa, and its increased detection in Veneto likely reflects the migration patterns from these areas. This subtype’s presence highlights the role of migration in shaping the local HIV epidemic and underscores the need for targeted public health strategies. It is also noteworthy how the circulation of non-B subtypes among newly diagnosed Italian subjects is increasing: the percentage of Italians in the total non-B population increased from 34.2% to 39.8% in the two periods; the percentage of Italian subjects with a non-B virus, therefore, increased from 25% to 32% on the total diagnoses in Italians.

In addition, recent geopolitical events, such as the Ukrainian war, have likely influenced migration patterns and, consequently, the epidemiology of HIV in Italy. The influx of refugees from Ukraine, a country where HIV-1 subtype A is predominantly prevalent, may have contributed to changes in subtype distribution in our region. Recent studies reflect this dynamic, including research by Wärnberg et al., which found that among the 98 Ukrainians tested for HIV in Sweden in 2022, the GRT was available for 24 individuals, with 23 showing subtype A6 [14]. Similarly, Załęski et al. reported a 78.7% prevalence of subtype A in people coming from Eastern European countries [15].

These observations highlight the need for targeted public health interventions and tailored ART strategies that consider the evolving epidemiological landscape. Enhanced surveillance and genotypic resistance testing are crucial for optimizing treatment regimens, particularly in regions with high migration and diverse HIV-1 subtypes. Understanding the social, demographic, and geopolitical factors driving these trends will be essential for effectively addressing TDRMs’ challenges and ensuring ART’s continued efficacy in diverse populations.

Similar findings have been shown in other studies, both in Europe and in the US, highlighting the impact of migration on local HIV epidemiology [16,17,18,19,20].

Our study noted a stable prevalence of TDRMs over time, which is reassuring, suggesting that current ART regimens effectively prevent the spread of resistant strains among newly diagnosed individuals. However, the persistent detection of TDRMs, including those against newer drug classes such as integrase strand transfer inhibitors (INSTIs), indicates ongoing challenges. Importantly, maintaining viral suppression is crucial not only for the health of the individual but also for public health, as achieving and sustaining an undetectable viral load through effective ART ensures that HIV cannot be sexually transmitted (U=U) [21,22]. This underscores the importance of tailoring antiretroviral therapy based on GRT, which is essential not only for optimizing individual treatment outcomes but also for ensuring the achievement and maintenance of an undetectable viral load.

Notably, our study found a low prevalence of NRTI mutations, which is decreasing compared to previous data [6,11]. This low prevalence of NRTI resistance supports the test-and-treat strategy not only with standard three-drug regimens but also with lamivudine (3TC)/DTG combinations. Conversely, we observed an increase in NNRTI mutations over time, exceeding 10% in the 2017–2024 period, with a higher percentage in non-B subtypes. These findings underscore the necessity to perform baseline GRT, with a view to a future simplification to long-acting treatment with cabotegravir-rilpivirine since NNRTI mutations have been associated with an increased risk of virological failure [23,24]. In addition, despite the European AIDS Clinical Society (EACS) guidelines and the DHHS guidelines suggesting that triple regimens with two NRTI and one NNRTI could be used as a first-line treatment, according to our results, this strategy should be avoided in our and similar settings, when the GRT is not available, due to the high prevalence of mutations for this class [5,25].

Regarding PIs, the prevalence of mutations remains anecdotal due to the high resistance barrier of this class. This could be reassuring for the clinicians since they could count on these drugs if a first-line regimen fails.

Finally, although still relatively low, the emergence of INSTI resistance mutations highlights the need for vigilance given the increasing use of these drugs in first-line regimens globally. Several recent reports show an increase in failure in people treated with INSTI, especially in Africa, with a higher percentage of people developing resistance to this class [26,27]. Bello et al., at CROI 2024, presented data about increasing DTG resistance in Malawi; they found that 15.5% of children in their cohort had DTG resistance [28]. Kingwara et al. found similar results, with 22% of DTG-resistance in ART-experienced PLHIV on DTG-based ART used as second-line ART and lower (8%) in ART-naïve adults using DTG for 1st-line ART, suggesting the risk of failing DTG may be higher in a background of pre-existing mutations to NRTIs in the regimen [29]. For all these reasons, it could be plausible that the detection of INSTI-resistance will increase, with potential repercussions in the clinical practice.

Regarding the specific mutation detected, our study highlights the significant issue of transmitted drug resistance. The persistence of E138A/K mutation across the study periods emphasizes the enduring challenge of NNRTI resistance, which affects the effectiveness of rilpivirine, highlighting the importance of performing GRT in naïve people living with HIV, especially with the view of future simplification to long-acting treatment with cabotegravir plus rilpivirine. Similarly, the detection of mutations like Y188L, F227L, and M230L, which also confer resistance to doravirine, indicates the spread of resistant strains capable of compromising initial treatment regimens.

Interestingly, few individuals showed mutations to NRTIs, with only two cases of K65R and three cases of M184V detected. This observation suggests that dual regimens incorporating lamivudine and DTG could still be viable starting options for this population.

Finally, the low occurrence of major protease inhibitor and integrase inhibitor resistance mutations in our ART-naive cohort suggests that these classes remain viable initial therapy options.

The disappearance of certain mutations impacting viral fitness can also be addressed, emphasizing the utility of advanced diagnostic methods. Next-Generation Sequencing (NGS) for minority populations or studies on peripheral blood mononuclear cells (PBMCs) could provide deeper insights into resistance patterns [30]. This approach is instrumental in detecting drug-resistance mutations in blood samples from ART-naive patients. Utilizing these sophisticated technologies enhances our detection capabilities and deepens our understanding of transmitted drug resistance, supporting more informed decision-making in HIV treatment strategies.

Our study has several limitations. It was conducted exclusively in the Veneto region of Italy, which may limit the generalizability of our findings to other areas or populations with different epidemiological profiles. The Genotypic Susceptibility Score (GSS) was not included, precluding a detailed assessment of the clinical implications of detected resistance mutations. Additionally, our dataset lacked specific information on the timing of entry into Italy and detailed histories of illness for HIV-positive migrants, which limits our understanding of the progression of infection relative to migration. Furthermore, not all participants underwent integrase genotypic resistance testing (GRT); only 70.8% and 81.3% were tested during the 2017–2019 and 2020–2024 periods, respectively. This incomplete testing might lead to an underestimation of the prevalence of integrase strand transfer inhibitor (INSTI) resistance mutations, potentially affecting our overall assessment of drug resistance. Lastly, the timeframe of our study (2017–2024) coincides with significant migration trends in the Mediterranean region. While we noted the influence of migration on the diversity of HIV-1 subtypes, the complex socio-political factors driving these trends were not explored, which might limit the contextual understanding of our findings.

Despite all the limitations, our study has many strengths. Firstly, the longitudinal design from 2017 to 2024 provides a comprehensive overview of trends in transmitted drug resistance mutations (TDRMs) and HIV-1 subtypes over an extended period. This allows for observing temporal changes and identifying emerging patterns, offering valuable insights into the evolution of HIV-1 resistance and subtype distribution. Our rigorous inclusion criteria ensure that all participants were antiretroviral drug-naive at the time of diagnosis, which is critical for accurately assessing the prevalence and impact of TDRMs in treatment-naive individuals. Focusing on a clearly defined population allows for more precise and meaningful conclusions regarding the initial treatment strategies for newly diagnosed PWH. The study’s findings align with broader trends observed in other regions and studies, reinforcing the relevance and applicability of our results. By situating our findings within the context of global HIV epidemiology, we contribute to a more comprehensive understanding of the factors influencing HIV resistance and subtype distribution.

## 5. Conclusions

In conclusion, our study affirms the dynamic nature of HIV epidemiology in Veneto, showcasing a diversification of subtypes and a stable but present level of drug resistance. The findings emphasize the importance of continuous monitoring of HIV subtypes and resistance patterns to tailor ART strategies effectively. While current therapies remain largely effective, the evolution of resistance, particularly to newer drug classes like INSTIs, requires ongoing surveillance and possibly anticipatory adjustments in treatment protocols. These insights not only aid in optimizing clinical outcomes but also contribute to the broader understanding of HIV dynamics in a global context. The study highlights the critical role of baseline resistance testing and adaptability in ART approaches to manage and mitigate the impact of HIV drug resistance.

## Figures and Tables

**Table 1:** Genotype and demographical characteristics of 762 people living with HIV, divided into 2017–2019 and 2020–2024.

Enrollment Periods	2017–2019		2020–2024	
PWH, *n*	414		348	
B subtype				
Total, *n* (%)	227 (54.8)		167 (48.0)	
	Italian	Foreigner	Italian	Foreigner
Male, *n*	171	25	118	19
Female, *n*	20	11	25	5
Total, *n*	191	36	143	24
Non-B subtype				
Total, *n* (%)	187 (45.2)		181 (52.0)	
	Italian	Foreigner	Italian	Foreigner
Male, *n*	53	63	56	64
Female, *n*	11	60	16	45
Total, *n*	64	123	72	109

**Table 2:** Immunological and virological baseline data were divided into 2017–2019 and 2020–2024.

	2017–2019		2020–2024	
	B-Subtypes	Non-B Subtypes	B-Subtypes	Non-B Subtypes
HIV viral load (cp/mL), median (IQR)	86,176 (17,424–383,000)	82,638 (12,725–299,021)	109,000 (24,200–505,741)	123,000 (24,991–820,442)
CD4+ (cells/mm^3^), median (IQR)	277 (80–545)	301 (99–481)	241 (71–492)	208 (74–432)
CD4 (%), median (IQR)	16.7 (8–26.3)	16.0 (7.1–27.0)	17.9 (8.0–26.0)	16.0 (7.6–23.0)
CD4 < 200, *n* (%)	39.0	38.9	47.3	47.8
CD4 < 350, *n* (%)	58.3	58.7	62.3	58.4

**Table 3:** Drug susceptibility prediction: single classes and combinations differentiated by B subtype and Non-B subtype population from 2017 to 2019 and 2020 to 2024.

	NRTI *n* (%)	NNRTI *n* (%)	PI *n* (%)	N + NNRTI *n* (%)	NRTI + PI *n* (%)	NNRTI + PI *n* (%)	Three Drug Classes *n* (%)	Total *n* (%)
B Subtype								
2017–2019	7 (3.0%)	25 (11.0%) *	1 (0.4%)	2 (0.9%)	0	0	0	35/227 (15.41%) *
2020–2024	4 (2.4%)	17 (10.2%) *	1 (0.6%)	0	0	1 (0.6%)	1 (0.6%)	24/167 (14.3%) *
Non-B Subtype								
2017–2019	1 (0.5%)	20 (10.7%) *	1 (0.5%)	0	0	0	0	22/187 (11.7%) *
2020–2024	2 (1.1%)	28 (15.5%) *	1 (1.1%)	3 (1.7%)	0	1 (1.1%)	0	35/181 (19.3%) *

* including mutations on position 138. NRTI: Nucleoside Reverse Transcriptase Inhibitors; NNRTI: Non-nucleoside Reverse Transcriptase Inhibitors; PI: Protease Inhibitors.

**Table 4:** Drug susceptibility prediction: single classes and combinations differentiated by Italian, European and Extra-European B-type population from 2017 to 2019 and 2020 to 2024.

B-Type Population	NRTI *n* (%)	NNRTI * n (%)	PI *n* (%)	N + NNRTI * n (%)	NRTI + PI *n* (%)	NNRTI * + PI *n* (%)	Three Drug Classes *n* (%)	Total *n* (%)
2017–2019								
Italians	5 (2.6%)	21 (11%)	1 (0.5%)	2 (1%)	0	0	0	29/191 (15.2%)
Europeans	0	1 (6.7%)	0	0	0	0	0	1/15 (6.7%)
Extra-Europeans	2 (9.5%)	3 (14%)	0	0	0	0	0	5/21 (23.8%)
2020–2024								
Italians	2 (1.4%)	17 (11.9%)	0	0	0	1 (0.7%)	0	20/143 (14.1%)
Europeans	0	0	0	0	0	0	0	0/4
Extra-Europeans	2 (10%)	0	1 (5%)	0	0	0	1 (5%)	4/20 (20%)

* including mutations on position 138. NRTI: Nucleoside Reverse Transcriptase Inhibitors; NNRTI: Non-nucleoside Reverse Transcriptase Inhibitors; PI: Protease Inhibitors.

**Table 5:** Drug susceptibility prediction: single classes and combinations differentiated by Italian, European and Extra-European Non-B-type population from 2017 to 2019 and 2020 to 2024.

NonB-Type Population	NRTI *n* (%)	NNRTI * n (%)	PI *n* (%)	N + NNRTI * n (%)	NRTI + PI *n* (%)	NNRTI * + PI *n* (%)	Three Drug Classes *n* (%)	Total *n* (%)
2017–2019								
Italians	0	6 (9.4)	0	0	0	0	0	6/64 (9.4)
Europeans	0	2 (11.8)	1 (5.9)	0	0	0	0	3/17 (17.7)
Extra-Europeans	1 (1.0)	12 (11.3)	0	0	0	0	0	13/106 (12.3)
2020–2024								
Italians	2 (2.8)	10 (13.9)	1 (1.4)	0	0	1 (1.4)	0	14/72 (19.5)
Europeans	0	4 (13.8)	0	1 (3.5)	0	0	0	5/29 (17.3)
Extra-Europeans	0	14 (17.5)	0	2 (2.5)	0	0	0	16/80 (20.0)

* including mutations on position 138. NRTI: Nucleoside Reverse Transcriptase Inhibitors; NNRTI: Non-nucleoside Reverse Transcriptase Inhibitors; PI: Protease Inhibitors.

## Data Availability

The original contributions presented in the study are included in the article and Supplementary Material, further inquiries can be directed to the corresponding author.

## Supplementary Materials

The following supporting information can be downloaded at https://www.mdpi.com/article/10.3390/v16091393/s1. Table S1: Drug susceptibility prediction: single classes and combinations are reported, expressed as absolute number of patients harbouring DRMs out of total of patients enrolled in each year.
