# Supplementary material for: Patterns of Transmitted Drug Resistance Mutations and HIV-1 Subtype Dynamics in ART-Naïve Individuals in Veneto, Italy, from 2017 to 2024
Source: Viruses. 2024 Aug 30;16(9):1393. doi: 10.3390/v16091393 (PMC11437434; doi:10.3390/v16091393)
Supplement: Supplementary file 1 [file viruses-16-01393-s001.zip › viruses-3127174-supplementary.pdf]

Table S1. Drug susceptibility prediction: single classes and combinations are reported, expressed as absolute number of patients harbouring DRMs out of total of patients enrolled in each year.

	NRTI[a] *n*	NNRTI[b] *n*	PI[c] *n*	N+NNRTI *n*	NRTI+PI *n*	NNRTI+PI *n*	3 drug classes *n*	Total *n*
**B-type**								
2017	3	9		2				14/84
2018	2	11	1					14/63
2019	2	5						7/80
2020	2	3				1		6/41
2021		6	1				1	8/46
2022	1	3						4/34
2023	1	4						5/38
2024		1						1/8
**NonB-type**								
2017		5						5/73
2018		8	1					9/62
2019	1	7						8/52
2020	1	3						4/29
2021		2	1					3/33
2022		6	1					7/48
2023	1	14	1	1	1			18/62
2024		3						3/9

[a] Nucleoside Reverse Transcriptase Inhibitors; [b] Non-nucleoside Reverse Transcriptase Inhibitors; [c] Protease Inhibitors.
